# The Midwifery Services Framework: The process of implementation

**DOI:** 10.1016/j.midw.2017.12.013

**Published:** 2018-03

**Authors:** Andrea Nove, Nester T. Moyo, Martha Bokosi, Shantanu Garg

**Affiliations:** a Novametrics Ltd, 4 Cornhill Close, Duffield, Derbyshire DE56 4HQ, United Kingdom; b International Confederation of Midwives, The Netherlands

**Keywords:** GIZ, Gesellschaft für Intenationale Zusammenarbeit (German international development agency), ICM, International Confederation of Midwives, M&E, monitoring and evaluation, MA, midwives’ association, MoH, ministry of health, MSF, Midwifery Services Framework, SRMNH, sexual, reproductive, maternal and newborn health, TWG, technical working group, UNFPA, United Nations Population Fund, USAID, United States Agency for International Development, WHO, World Health Organisation, Midwifery, Health workforce, Sexual, reproductive, maternal and newborn health, Human resources for health, Sustainable development goals, Universal health coverage

## Abstract

In 2015, the International Confederation of Midwives launched the Midwifery Services Framework: a new evidence-based tool to guide countries through the process of improving their sexual, reproductive, maternal and newborn health services through strengthening and developing the midwifery workforce. The Midwifery Services Framework is aligned with key global architecture for sexual, reproductive, maternal and newborn health and human resources for health, and with the recommendations of the 2014 Lancet Series on Midwifery. This second in a series of three papers describes the process of implementing the Midwifery Services Framework: the preparatory work, what happens at each stage of implementation and who should be involved at each stage. It gives an idea of the scale of the task, and the resources that will be required to implement the Midwifery Services Framework in a given country context. The paper will be of interest to health policy-makers, development partners and professional associations in countries considering different approaches to strengthening their sexual, reproductive, maternal and newborn health services, and it will help them to decide whether and when either full or partial/staged implementation of the Midwifery Services Framework will be an appropriate initiative to address identified deficits in their specific context, given the current and projected availability of resources.

## Highlights

• Investing in the midwifery workforce is an effective strategy for improving outcomes.
• The Midwifery Services Framework (MSF) provides guidance on how to do this in a systematic way.
• This paper explains how the MSF is implemented.
• It is a multi-stakeholder, country-led process with several stages.
• Commitment and resources from a range of stakeholders is necessary.

## Introduction

In March 2015, the International Confederation of Midwives (ICM) launched the Midwifery Services Framework (MSF), and since then implementation of the MSF has begun in eight countries. The MSF (ICM, 2015) was a response to global calls for improved health outcomes via investment in the health workforce (United Nations, 2016, WHO, 2016a), and was based on compelling evidence that investment in midwifery is a cost-effective way to improve sexual, reproductive, maternal and newborn health (SRMNH) outcomes (The Lancet, 2014; UNFPA et al., 2014), but that in some countries midwifery is not a particularly valued profession and has not received appropriate investment (Filby et al., 2016, WHO, 2016b).

The development of the MSF was led by ICM in collaboration with partner organisations including the World Health Organisation (WHO) and United Nations Population Fund (UNFPA). It is a tool which assists countries to operationalise the concept of strengthening the midwifery profession to enable it to meet the need for SRMNH care. The planning and implementation of a process of strengthening an entire profession is a complex undertaking (WHO, 2011) and therefore requires a significant investment of time and other resources, as well as political will (Gualda et al., 2013, Polishook and Cortese, 2000, WHO, 2016c), understanding of legal, regulatory and governance frameworks (Barbazza et al., 2015, Verani et al., 2011), and logistical and technical expertise in key areas such as professional education and training (WHO, 2016c).

Because the process of strengthening a profession may be resource-intensive and requires a long-term commitment, it is helpful for those charged with implementing the MSF or considering whether it is an appropriate tool to apply in their country to have a broad understanding of the practical implications: what they will need to do, who will need to be involved, and what resources will be required. This, the second in a series of three papers about the MSF, aims to provide this understanding. The first paper explains how and why the MSF came into being. The third paper describes the lessons learned so far from the first countries to begin implementation of the MSF.

## Definitions and key concepts

In considering whether or not the MSF might be useful in a particular country, it is important first to understand some definitions and key concepts about midwives and midwifery:

### Guiding principles of midwifery

Professional midwifery care (including front-line emergency obstetric care) should be available, accessible, and acceptable to all women, and should be of good quality, including being sensitive to gender and culture and respectful of service users. Midwives should provide evidence-based services along the entire continuum of care from adolescence and pre-pregnancy through pregnancy care, childbirth and the postnatal period, including family planning and safe abortion care (PMNCH, 2011). They should be able to provide services in the full range of settings from home, through primary health care facilities to referral hospitals.

To achieve the above, midwives must be educated and regulated according to global standards (ICM, 2013a, ICM, 2011a), and operate within a functioning health system including other cadres of health worker such as obstetricians and nurses (UNFPA et al., 2014).

### What is a midwife?

A midwife is a responsible and accountable health professional who works in partnership with women to provide the necessary support, care and advice from the point at which a woman or girl becomes sexually active, through pregnancy, childbirth and the postnatal period. Midwives are skilled birth attendants as defined by WHO (WHO, 2004) and can conduct births on their own responsibility as well as provide care for the newborn. The role includes health counselling and education for women, their families and the wider community.

Not all midwives have the job title ‘midwife’. For example, some countries have nurse-midwives who either have nursing responsibilities in addition to their midwifery responsibilities, or who pursued a career in midwifery after initially qualifying as a nurse. In this paper, the word ‘midwife’ is used to refer to any health worker who fits the description in the preceding paragraph, regardless of job title or the educational pathway they took to become qualified.

### Midwifery competencies

ICM has defined seven midwifery competencies (ICM, 2013b), which form the basis of the MSF and are summarised as follows: (1) knowledge and skills from obstetrics, neonatology, the social sciences, public health and ethics that form the basis of high quality, culturally relevant, appropriate care for women, newborn infants, and childbearing families; (2) health education and services to promote healthy family life, planned pregnancies and positive parenting; (3) antenatal care, including early detection and treatment or referral for complications; (4) care during labour; (5) postpartum care for women; (6) care for the newborn up to two months of age; and (7) abortion-related care as appropriate within the applicable laws, regulations and protocols.

## Stages of the MSF

**Fig. 1:**
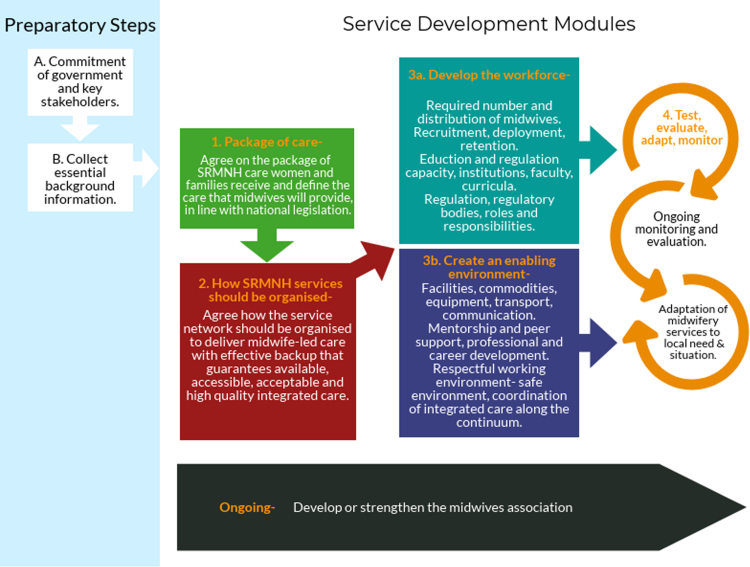
Stages of implementation of the ICM Midwifery Services Framework.

Prior to commencing implementation of the MSF, two preparatory steps take place: (1) securing the commitment of government and other key stakeholders, and (2) collection of essential background information. The MSF itself consists of four modular service development steps: package of care, organisation of SRMNH services, workforce and work environment, and ongoing monitoring and evaluation (M&E). Underpinning all of these activities is the development or strengthening of the national midwives’ association (MA). Thus, in total there are seven steps, illustrated in Fig. 1 and described below.

### Preparatory step A: Secure commitment of key stakeholders

Because the MSF is a country-led process, it can only be successful if those responsible for leading the process commit to providing the necessary leadership and/or resources (human and financial). This means that the appropriate department(s) within the national ministry of health (MoH) should understand the MSF, agree that it aligns with the country's broader SRMNH policies and strategies, and commit to supporting the process. It is recommended that other key stakeholders such as UN agencies, professional associations (including those from relevant professions other than midwifery), funders and service providers should also be willing to support the process. From these stakeholders, a lead organisation is selected. To fulfil this role effectively, it is important for the lead organisation to: have a base in the country, have a maternal health mandate, have a respected track record, be willing to work in partnership with the MoH, the professional MA and other relevant stakeholders, and have the necessary management, administrative and financial functions. The organisation is identified through discussions between ICM, the MoH, the MA and other stakeholders.

### Preparatory step B: Collect essential background information

This step is essentially a situation analysis for SRMNH services, which ensures that decisions and actions taken during the service development steps are evidence-based and tailored to the country context. The information falls into four categories: (1) the anticipated number and geographical distribution of pregnancies over the next 10 years, (2) population characteristics such as age profile, urbanisation, adolescent pregnancy rate, wealth distribution, (3) health system characteristics, e.g. types, numbers and locations of health facilities providing SRMNH services, relative size and scope of public and private health sectors, availability of drugs, equipment and other essential supplies, SRMNH policy environment, and (4) SRMNH workforce characteristics, e.g. availability, geographical distribution (e.g. using analysis based on geographical information systems), age and gender profile, migration patterns, deployment mechanisms, education pipeline. Guidance exists for locating, collating and analysing the information in a way that aligns with the MSF process (UNFPA and WHO, 2015).

### Ongoing activity: Set up and/or develop and strengthen the midwives’ association (MA)

Together with education and regulation, MAs form the foundation of a strong midwifery profession (ICM, 2017). A strong professional association is nationally representative, sustainable, member-led and has the capacity to carry out a range of functions as appropriate to the country context, such as quality assurance, advocacy for the needs of service users and midwives, and contribution to relevant legislation (McQuide et al., 2007). The sustainability of activities initiated under the MSF is dependent on a strong national MA, so part of the MSF process is to assess and, where needed, improve the capacity of the MA in each country. An initial ‘gap analysis’ can identify areas that require strengthening (ICM, 2011b), and ICM can provide support to address the gaps. If there is no MA in the country, ICM can provide advice and support to set one up.

### Service development step 1: Agree on the package of SRMNH care that women and their families should receive and define the services that midwives will provide

The objective of this step is to define: (a) the package of SRMNH care to which women and newborns are entitled within a defined, agreed timeline, and (b) the elements of this package of care that should be delivered by midwives (as opposed to other cadres of health worker). Many countries already have a ‘minimum benefits package’ which defines the entitlement to SRMNH care (UNFPA et al., 2014), which can be used as a basis for reaching agreement on (a), and likewise many countries have an agreed and documented scope of practice for midwives which may provide the answer to (b). However, it is helpful to assess the extent to which these documents align with global standards (ICM, 2013b, WHO, 2010) and are based around the needs of women and their families, and if they do not, to develop a vision for the future with short-, medium- and long-term goals.

### Service development step 2: Discuss how SRMNH services should be organised to deliver midwife-led care with effective back-up

Although most countries have a similar set of health worker cadres in their SRMNH workforce (UNFPA et al., 2014), the way in which SRMNH services are organised varies considerably. At one end of the continuum is an entirely midwife-led model of care under which midwives are the lead health professional for pregnant women unless there is a complication which requires referral to a physician, and at the other end are physician-led models under which midwives perform a role akin to an obstetric nurse or an auxiliary cadre – and there are many other arrangements in between these two extremes (DeVries et al., 2001, Klopper and Uys, 2013). Even under the heading of ‘midwife-led care’, there is a variety of models, including caseload, team, community-based and facility-based midwifery (Sandall et al., 2016). Based on evidence of the efficacy of midwife-led care (Sandall et al., 2016), the MSF advocates a midwife-led model, which for some countries will require significant reorganisation of SRMNH services whilst maintaining full integration of these services within the broader health system.

### Service development step 3: Develop the workforce and create an enabling environment

This step is divided into two linked and concurrent stages, one relating to the workforce and the other to the workplace. The ‘workforce’ stage aims to establish how to meet the country's current and future requirements in terms of the availability, education and regulation of midwives and how these can be organised to meet the needs of women and their families. During this step, reference may be made to established guidance and benchmarks for estimating the number of midwives needed (WHO, 2016a, WHO, 2005), bearing in mind that the needs of individual countries will vary according to the prevailing demographic and epidemiological conditions (ten Hoope-Bender et al., 2017). Reference should also be made to established global standards for midwife education and regulation (ICM, 2013a, ICM, 2011a).

The ‘workplace’ stage recognises that the workforce can only meet the needs of the population if there is an enabling work environment, i.e. one with the equipment, supplies, infrastructure and referral system needed to provide high-quality and respectful care, and in which workers are motivated by a safe, collaborative and supportive work environment. During this stage, consideration is given to how to ensure such an enabling environment.

### Service development step 4: Test, monitor, evaluate and adapt midwifery services

At an appropriate stage in the process (often alongside service development steps 1 and 2) stakeholders are asked to define what successful implementation would look like, and based on this definition, to agree on time-bound targets to be achieved as a result of MSF implementation. This leads to the selection of relevant indicators to allow monitoring of progress towards the targets, which in turn require the collection of monitoring data, either on an ongoing basis or at agreed intervals as appropriate to the indicator. The data collected during preparatory step B may be used to reflect the baseline situation for each indicator, so that future progress can be measured against this baseline.

The ongoing M&E involves subjecting the decisions and actions taken during steps 1–3 to scrutiny, with a view to determining whether or not they produced the intended results. This is an ongoing process rather than a single activity, because the MSF is designed to provide needs-based SRMNH services, and the level and type of need for these services changes over time. Reacting to change and anticipating future change is therefore vital for successful implementation of the MSF. In addition to monitoring progress towards the agreed targets, a thorough (and ideally independent) evaluation of successes and failures during steps 1–3 is also recommended. These activities will help to ensure accountability, and to ensure that lessons are learned for the future development of SRMNH services.

If the country already has an M&E mechanism for health outcomes and/or the health workforce, M&E of the MSF can be incorporated into it. Otherwise, a bespoke mechanism will need to be designed, based on established guidelines such as those produced by the World Health Organization (WHO, 2009a, WHO, 2009b). ICM is currently working to design an M&E template for the MSF, which countries can adapt to the country context.

## The practicalities of implementing the MSF

From a practical perspective, MSF implementation involves four broad phases, each of which involves ongoing stakeholder engagement and dialogue: (i) introductory meetings, (ii) data collection and workshop preparation, (iii) country assessment workshop, (iv) service development. Table 1 maps these phases alongside the MSF steps to show at which phase each MSF step occurs. The length of time taken to implement each phase varies by country. As a minimum, countries considering MSF implementation should allow 2 months to set up the introductory meetings, then an additional 3 months to do the data collection and workshop preparation. No country has yet completed the service development phase so it is impossible to state how long it takes, but it is anticipated that it could take 3–5 years for a country needing to complete all of the steps.

**Table 1:** Implementation phases mapped onto steps of the MSF.

**Implementation phase**	**MSF steps**	**Ongoing activity**
i. Introductory meetings	Preparatory step A: commitment of government and key stakeholders	
ii. Data collection&workshop preparation	Preparatory step B: data collection	Association strengthening, stakeholder feedback loop
iii. Country assessment workshop	Service development step 1: package of care	
	Service development step 2: organisation of services	
iv. Service development	Service development step 3: workforce and workplace	
	Service development step 4: test, evaluate, adapt, monitor	

### Implementation phase (i): Introductory meetings

A series of introductory meetings takes place, to ensure connections between all relevant country stakeholders. The main objectives are that those attending the meetings will gain a clear understanding of the MSF and a decision will be made either to proceed (either fully, or in a partial/staged way), or not to do so. If the decision is to proceed, an MoH representative is nominated to lead the process, and the MoH expresses an openness to review the SRMNH system and take actions to improve it as these are identified during the MSF process. Under the leadership of the MoH, other stakeholders usually commit to make financial and/or technical contributions to MSF implementation, a chairperson is appointed, nominations are made for people to join the MSF implementation taskforce, and a taskforce secretariat is appointed. Plans are also made for the next steps of implementation, including who will be involved in preparatory step B (data collection).

### Implementation phase (ii): Data collection and workshop preparation

If needed, ICM provides template data collection tools for country stakeholders to populate. It may not be possible to populate the tools fully in advance of the country assessment workshop (in fact, deliberation at the workshop will almost certainly be required for some data items), but the more complete they are before the workshop, the easier it will be to work through the workshop activities. At the same time, preparations are made for the workshop, during which the agenda, participant list and logistical arrangements (e.g. venue) are agreed.

### Implementation phase (iii): Country assessment workshop

This three-day workshop includes a number of presentations and structured activities facilitated by ICM and other partners, with a view to reaching consensus on key issues, including: (1) definition of the package of care that women and families should receive, and which elements of this package should be delivered by midwives, (2) how the SRMNH system should be organised to deliver effective coverage of midwifery care, (3) what challenges the country faces in terms of the SRMNH workforce and working environment, (4) the priority order in which challenges should be addressed, and (5) which organisations and individuals should be charged with responsibility for addressing the challenges and for M&E of the MSF process. Based on these decisions and under the leadership of the MoH, technical working groups (TWGs) are formed, and given responsibility for one or more specific aspects of MSF implementation.

After the workshop, ICM attends debriefing meetings with the MoH and other relevant partners, during which TWG terms of reference and action plans are drafted. ICM also drafts a workshop report so that key issues and decisions are documented for future reference by all stakeholders.

### Implementation phase (iv): Service development

Based on the workshop report, terms of reference and action plans, the TWGs take forward the agenda agreed at the workshop. Their main focus is on developing the SRMNH workforce and working environment, but TWGs may also be charged with other relevant activities such as M&E data collection or completing any unfinished activities under service development steps 1 and 2. This phase of the process is entirely country-led, but if any TWG requires technical expertise that is not easily available in country, ICM can use its global network of experts to provide the necessary advice and/or practical support.

During this phase, ICM carries out a programme of work with the national MA to strengthen it, so that the MA can support the introduction or mainstreaming of the midwife-led model of care that is central to the MSF. These strengthening activities will help the MA to be a competent and credible advocate for the midwifery profession. Many of the TWG activities will require long-term investments of time and resources which may span successive governments and/or funding cycles, and there is therefore a risk that the political will to implement the MSF will wane over time and the process will stall. To mitigate this risk, effective engagement with policy-making and persistent advocacy efforts from the MA will be needed to keep the MSF high on political, funder and health system agendas for as long as necessary to achieve its objectives.

## Funding the MSF

Seed funding for introductory meetings and country workshops was initially provided by the Bill and Melinda Gates Foundation (reference OPP 1127391_ICM). It was anticipated that this would cover the costs of the introductory meetings and workshops for 3–4 countries. Due to the interest from additional countries, ICM initiated a cost-sharing arrangement. Under this arrangement, a consortium of partners has tended to share the costs of the country workshops, including lead organisations (e.g. UNFPA, Jhpiego) and funders (e.g. USAID, GIZ).

## Conclusion

The MSF is designed to be applicable in all contexts; its modular nature means that countries can choose which parts to implement and where the main focus should be, according to the national context. The recommended process is clearly documented in guidance notes (ICM, 2015), so that countries with strong professional associations and the political will to make improvements to SRMNH via investment in midwifery can apply the ICM guidance independently. Other countries will need support from ICM and partners to follow the process described in this paper, which requires sustained commitment of time and resources from a range of stakeholders. Experience to date suggests that successful and conscientious implementation will result in a well-supported, strong, sustainable, needs-based and evidence-based SRMNH care system with leadership from all relevant health workers including midwives.
